# Identification of New Chickpea Virus and Control of Chickpea Virus Disease

**DOI:** 10.1155/2022/6465505

**Published:** 2022-05-28

**Authors:** Zihui Cun

**Affiliations:** College of Life Sciences, Wuhan University, Wuhan, Hubei, China

## Abstract

**Objective:**

The objective of the study was to discuss the classification, virus characteristics, detection methods, and control measures of chickpea virus, with an aim to provide a theoretical basis for identification of new chickpea virus and control of chickpea virus disease.

**Methods:**

The domestic and foreign studies were reviewed, and the virus coat protein or nucleic acid sequence was identified by immunological and molecular diagnostic techniques.

**Results:**

There were 14 main types of chickpea viruses attacking, and seven Luteoviridae viruses were reported, namely, chickpea chlorotic stunt virus (CpCSV), bean leafroll virus (BLRV), beet western yellows virus (BWYV), soybean dwarf virus (SbDV), cotton leafroll dwarf virus (CLRDV), cucurbit aphid-borne yellows virus (CABYV), and phasey bean mild yellows virus (PhBMYV). The family Geminiviridae includes chickpea chlorotic dwarf virus (CpCDV), chickpea chlorosis virus (CpCV), chickpea redleaf virus (CpRLV), chickpea yellows virus (CpYV), and mastrevirus. The family Nanoviridae is dominated by the faba bean necrotic yellows virus (FBNYV). The family Bromoviridae includes cucumber mosaic virus (CMV) and alfalfa mosaic virus (AMV).

**Conclusion:**

At present, there are mainly 12 types of viruses infecting chickpeas, which are transmitted by leafhoppers or aphids and are associated with symptoms such as yellowing, chlorosis, and stunted pod development, resulting in serious yield loss. Correct use of various molecular diagnostic tools to detect and identify chickpea virus can accurately assess chickpea virus infection and provide a basis for the prevention and treatment of chickpea virus disease.

## 1. Introduction

Chickpea (*Cicer arietinum* L.) belongs to the genus Chickpea in the family Leguminosae, with a long history of cultivation, and first appeared in western Asia and the Near East. It is now mainly distributed in the Mediterranean, Asia, Africa, and America [1]. In China, chickpea is mainly grown areas with an altitude of about 2000–2700 meters, including Gansu, Qinghai, Xinjiang, Shaanxi, and Shanxi provinces. Chickpea is drought- and cold-tolerant as well as barrenness-resistant, and its well-developed root system, large and numerous root nodules, and strong nitrogen fixation capacity facilitate soil and water conservation and ecological management (Dilizati-Dolikun, 2019). Chickpeas are high in nutritional value, rich in amino acids, vitamins, dietary fiber, and beneficial unsaturated fatty acids and are an important source of nonanimal protein [2, 3].

In recent years, the frequent and extensive occurrence of chickpea virus disease has been identified as one of the major causes of 30%–50% yield reduction in chickpea. Thus, the response to the global food crisis can be substantially benefited through further research of symptom characteristics of virus diseases, identification of virus infestation, and formulation of effective control measures. Currently, 12 viruses infesting chickpea have been found, which are divided into two groups. The first group includes CMV and AMV, mainly transmitted by aphids and to a lesser extent by seeds and mainly attacks leaves, causing mosaic or mottled symptoms. The second group includes FBNYV, BLRV, BWYV, CpCSV, and CpCDV, which are mainly found in the Middle East and West Africa and are transmitted by aphids or leafhoppers. They will cause yellowing, stunting, and poor pod development in chickpea, resulting in yield reduction. Currently, there are no detection and identification methods with satisfactory outcomes for the second group of viruses [4–7].

The paper reviews the classification and virus characteristics of domestic and overseas chickpea viruses as well as chickpea virus detection methods and control measures, with a view to providing a theoretical basis for identification of new chickpea viruses and control of chickpea virus diseases.

## 2. Virus Characteristics of Chickpea

### 2.1. Luteoviridae Viruses

To date, seven Luteoviridae viruses have been reported [8], namely, chickpea chlorotic stunt virus (CpCSV), bean leafroll virus (BLRV) [6, 9], beet western yellows virus (BWYV) [6, 8, 10], soybean dwarf virus (SbDV), cotton leaf roll dwarf virus (CLRDV), cucurbit aphid-borne yellows virus (CABYV), and phasey bean mild yellows virus (PhBMYV), and the details are given in Table 1. The viral particles of Luteoviridae are isometric symmetric icosahedral, hexagonal in shape, without envelope, 25–30 nm in diameter. The viral particles are composed of 28% nucleic acid and 72% protein, and the molecular mass of shell protein is 21–23 kDa [31]. All seven chickpea viruses are RNA viruses with similar structures, mainly distributed in temperate, subtropical, and tropical regions, transmitted by aphids in a persistent manner. None of them can be transmitted by mechanical inoculation. Most of the chickpea Luteoviridae family viral disease symptoms are similar, and there is a serological relationship between the viruses.

CpCSV, BLRV, BWYV, and SbDV may cause plant dwarfing, yellowing, reddening of leaves, no pods or poor pod set, and significant yield reduction, thus causing significant economic losses [17, 19]. Currently, most of these viruses have not been well identified [23, 31]. CLRDV and CABYV belong to the Luteoviridae potato leafroll virus and are rapidly transmitted by aphids in a cyclic-persistent manner. CLRDV was initially identified as an infestation of cotton, causing stunting, leaf curling, dense green leaves, yellowing of veins, brittle leaves, reduced flowers and boll size, and plant sterility in some cases, which can lead to yield losses of up to 80% in some susceptible varieties [5]. CLRDV virus may also infect chickpea, causing symptoms similar to those of cotton (reddening of leaves, shortening of internodes, and stunting and browning of the bast) [21, 24, 32]. Similar symptoms of chickpeas can also be seen in CABYV infestation. At the molecular level, PhBMYV exhibits the most similar nucleic acid sequence to CABYV and is continuously transmitted by aphids; however, PBMYV infestation of chickpea shows less pronounced symptoms, including mild growth retardation, dwarfing of leaves and branches, and reddening or yellowing of leaves (Sharman et al., 2016) [22].

### 2.2. Geminiviridae Viruses

The family Geminiviridae is persistently transmitted by leafhoppers and cannot be transmitted by mechanical inoculation [27, 31], including chickpea chlorotic dwarf virus (CpCDV), chickpea chlorosis virus (CpCV), chickpea redleaf virus (CpRLV), chickpea yellows virus (CpYV), and mastrevirus [12, 27]. The primary effect of Geminiviridae infestation of chickpea is plant yield reduction, with infection during early growth causing near crop failure and infection during flowering with yield losses of 75–90% [33], but this group of viruses has a narrow natural host range and is highly immunogenic. Geminiviridae virus particles have a duplex structure, consisting of two incomplete icosahedra without an envelope and a genome consisting of 2.6 to 2.8 kb of single-stranded DNA [12, 34, 35].

In 1993, Horn et al. first identified CpCDV, a virus of the family Geminiviridae, in India [25], and 19 types of CpCDV (CpCDV-a to s strains) have been reported. Then, CpCDV viruses have also been identified in the Middle East, Africa, South Africa, North Africa, South Asia, and the Arabian Peninsula [13, 18, 26–28, 36–41]. Three CpCDV species of chickpea chlorotic dwarf Sudan virus (CCDSV), chickpea chlorotic dwarf Pakistan virus (CCDPV), and chickpea chlorotic dwarf Syria virus (CCDSV) can infest chickpea [6]. CpCV and CpRLV were identified in New South Wales in 2002. CpYV and mastrevirus are only present in Australia [4, 12, 27].

### 2.3. Nanoviridae Viruses

The family Nanoviridae is dominated by the genus faba bean necrotic yellows virus (FBNYV), which has a wide range of virus hosts and has been identified to infest more than 50 species of plants (mainly belonging to the legume family) [15, 42]. This group of viruses is transmitted by aphids in a persistent manner and cannot be transmitted by mechanical inoculation, and the effective vectors of FBNYV are aphids and pea aphids [11]. The virus particles consist of small icosahedral particles 17–20 nm in diameter, single-stranded circular DNA viruses with multicomponent genomes, each with a molecular size of about 1 kb ([15], [43, 44]), and a viral capsid composed of proteins with a molecular weight of about 20 kDa [45]. FBNYV was first isolated from snap beans near Latakia, Syria, and can cause severe yield losses and crop failure [15, 29, 30]. FBNYV is taxonomically closely related to *Astragalus sinicus* milk vetch dwarf virus (MDV). It can damage the bast, and chickpea is severely stunted and slightly discolored after infestation, with young leaves curled, incompletely developed, thickened, and brittle leaves, and interveinal symptoms such as faded green spots, smaller young leaves that cup upwards, while mature leaves curl downwards, and stunted new shoots, leaves, and flowers [15, 43, 44]. Interveinal yellowing and necrosis appear about 3-4 weeks after infection, and plants die within about 5–7 weeks after infection [46].

### 2.4. Bromoviridae Viruses

The family Bromoviridae, which includes cucumber mosaic virus (CMV) and alfalfa mosaic virus (AMV), is distributed in temperate and tropical areas with an extremely wide host range [47]. The viruses are transmitted nonpersistently by aphids and can be transmitted mechanically by inoculation. CMV particles consist of three isosymmetric icosahedra, each of uniform size, with a diameter of about 29 nm and no envelope. Bromoviridae viruses are RNA viruses, with RNA 1 and RNA 2 each encapsulated in one particle and RNA 3 and RNA 4 in one particle. The capsid protein consists of a polypeptide with a molecular mass of 24 kDa [20, 48]. The nucleic acid accounts for about 18% of the weight of the viral particle and the capsid protein for 82%. In contrast, the viral particle of AMV consists of a multicomponent granule, elongated or bacillary in shape, with a diameter of 18 *μ*m and lengths of 58 *μ*m, 49 *μ*m, 38 *μ*m, and 29 *μ*m, respectively; the other is a subspherical body with a diameter of about 18–20 *μ*m. The nucleic acid accounts for about 18% of the weight of the viral particle, and the capsid protein occupies 82% [49–51].

CMV was first reported by Doolittle and Jagger in 1916 (Doolittle, 1916; [52]), while AMV was first identified in the United States in 1931 [53]. Symptoms of chickpea infestation by Bromoviridae include pale green or yellowing mottling (phloem), distorted and deformed leaves or petioles, small, slightly crinkled diseased leaves, severe leaf recoil, and gradual yellowing of the lower leaves of the diseased plant, i.e., phloem or mottling symptoms. The main symptoms of CMV infestation are plant stunt, leaf malformation, and mosaic [54]. In contrast, after AMV infestation of the plants, the host leaves showed typical symptoms such as phloem, crinkling, dwarfing, curling, mottling, necrosis, fading green, and yellowing [55, 56].

## 3. Detection Technology of Chickpea Virus

Chickpea virus disease seriously compromises the yield of chickpeas. Accurate and rapid identification and detection of the chickpea virus can effectively mitigate the harm. In the early stage, the virus was mainly identified by growth detection or direct observation, and the infecting virus was identified directly through observation of the symptoms of infected plants. The method is simple and cost-effective but susceptible to environmental factors. Given the similar symptoms of chickpea virus infestation, accurate identification of the viruses is complicated. Immunological and molecular diagnostic techniques can accurately identify viral coat proteins or nucleic acid sequences, thereby accurately identifying virus species. The techniques currently used in the detection of chickpea virus include serological or immunological detection, polymerase chain reaction, and high-throughput sequencing technology.

**Figure 1:**
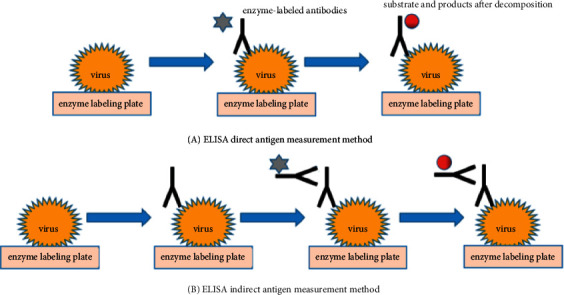
Enzyme-linked immunosorbent assay.

Serology technology is an immunological technology, which uses antigen-antibody-specific binding in vitro for identification. This method is simple to operate with accurate results and is widely used in plant virus detection. According to the principle of color development, it is divided into enzyme-linked immunosorbent assay (ELISA) (see Figure 1) and immunocolloidal gold assay [57, 58]. ELISA can determine the affinity of phage to CpCDV-CP [14], and ELISA or tissue blot immunoassay (TBIA) is used to detect chickpea virus in infected tissues, to accurately distinguish FBNYV from Luteoviridae (or Geminiviridae) [15]. However, the ELISA is cumbersome and requires special equipment. Therefore, methods such as A protease-linked adsorption (SPA-ELISA), dot immunosorbent (DIBA), direct tissue plaque immunoassay (IDDTB), voltammetric enzyme-linked immunoassay, and rapid ELISA have been developed. No special instruments or apparatus is required in the immunocolloidal gold method, which facilitates rapid field diagnosis and port quarantine. It can rapidly detect soybean mosaic virus [3, 59, 60], but cannot accurately quantify plant viruses. Serological techniques usually require more than two methods for verification as one method is less accurate [61].

**Figure 2:**
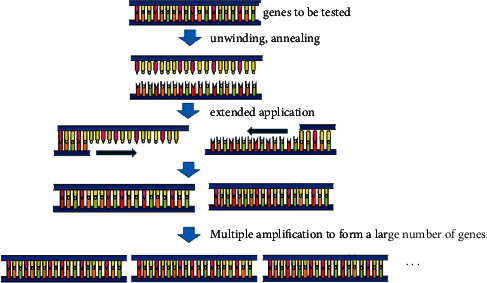
PCR principle.

**Figure 3:**
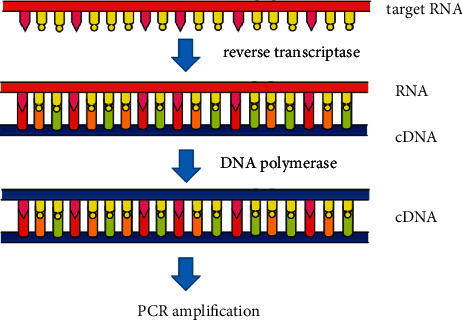
RT-PCR principle.

Molecular diagnostic techniques allow the determination of viral nucleic acid sequences and thus the identification of viral species. The techniques are simple, sensitive, specific, and effective for the detection of low-load viruses. Polymerase chain reaction (PCR) is effective in detecting and identifying DNA viruses (e.g., Dictyoviridae viruses) [57] (Figure 2), and reverse transcription-polymerase chain reaction (RT-PCR) is effective in detecting RNA viruses (e.g., Luteoviridae viruses) [57, 62] (Figure 3). Both methods use specific primer pairs targeting viral gene regions for PCR or RT-PCR amplification and identification or detection of chickpea viruses by sequence analysis. The Iranian CpCDV-F isolate was amplified and sequenced by the PCR method and was shown to be closely related to CpCDV-A and CpCDV-F isolates [63]. In addition, the genomic DNA of FBNYV isolates could be specifically detected by the immunocapture (IC)-PCR method [42].

The aforementioned methods can detect plant viruses at a pg or even fg level, but differences are found in sensitivity between methods. Rowhani et al. used RT-PCR, IC-PCR, and ELISA to detect ApMV (apple mosaic virus), PDV (prune dwarf virus), PNRSV (prunus necrotic ringspot virus), GFLV (grapevine fanleaf virus), and CLRV (cherry leafroll virus) and found the highest sensitivity of RT-PCR, followed by IC-PCR, while ELISA was the least sensitive (Table 2). However, the sensitivity of various assays for chickpea virus is marginally explored [64].

## 4. Control Methods of Chickpea Virus

**Figure 4:**
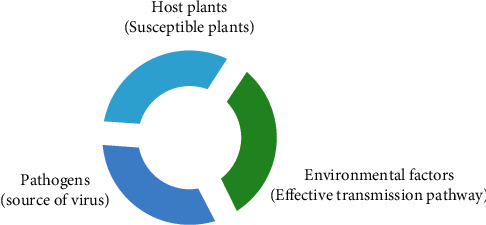
RT-PCR principle.

The control of plant viral diseases requires consideration of the “plant host-pathogen-environmental factors” and their interconnection to effectively control virus infections and losses (see Figure 4). Currently, no effective methods are available to inhibit virus reproduction in plants. The most effective control measures rely on virus epidemiology, the use of integrated disease management methods in agricultural practices [6], and increased virus detection, to improve the virus prevention [65].

### 4.1. Enhancing Chickpea Resistance

Enhanced disease resistance in chickpea can reduce the damage caused by viruses, such as the selection of disease-resistant plants and the use of various factors to induce the production of disease resistance [65]. Currently, there are three methods to enhance disease resistance in chickpea as follows.

The first is to screen disease-resistant varieties. Comparative trials were conducted on different chickpea varieties to screen out different varieties with high fresh grass yield, high seed yield, strong adaptability, drought, and disease resistance to improve the disease resistance of chickpea. For example, the varieties FLIP94-68C, FLIP94-93C, and FLIP94-80C with excellent traits were screened in the Gansu area, which have strong disease and drought resistance and can be promoted in arid or semiarid areas [66].

The second is crossbreeding, where genetic recombination generates new genotypes and screens for new superior traits. Different chickpea varieties differ greatly in their resistance to BLRV, and crossing between more distantly related varieties to screen for resistant monocultures in the progeny can significantly increase the proportion of resistant plants [16].

The third is transgenic breeding, where antisense sequences of target virus gene fragments are transformed into plants to trigger the host plant to develop resistance to this virus through a gene silencing mechanism. For example, transgenic fava beans resistant to FBNYV infection effectively reduce the risk of plant infection with this virus and reduce disease losses [15].

### 4.2. Reduction of Transmission Routes

The control of the large-scale occurrence of virus diseases requires both intervention in the plants and reasonable ways to cut off the virus transmission channels and minimize the damage caused by virus diseases. Twelve chickpea viruses are transmitted by leafhoppers or aphids, so controlling the number of leafhoppers or aphids can effectively reduce the damage caused by chickpea virus diseases. Therefore, aphids and leafhoppers should be trapped and deterred regularly to lower the risk of plant infection; diseased plants should be cleaned in time to eliminate the pathogen, and operators, farm tools, and machinery should be disinfected to eliminate potential viruses [6, 65].

### 4.3. Integrated Disease Management Approach

Measures such as controlling the sowing period, sowing volume and row spacing, inducing disease resistance early in the growing season, and cultivating disease-resistant and early-maturing varieties are effective in reducing virus infection in the field. Pretreatment of seeds with 1-2 insecticide sprays or broad-spectrum insecticides before planting can be effective in reducing virus transmission and virus disease incidence [6]. Virus disease control measures need to be field researched and adapted to local conditions to identify specific measures for chickpea virus disease control in a particular region. For example, in northern Sudan, delaying chickpea planting by 3-4 weeks, shortening the irrigation period, and using resistant chickpea varieties significantly reduced the incidence of CpCDV in chickpeas [67]. In Egypt, late autumn planting, postemergence aphicide sprays (two sprays early in the season when the virus disease incidence peaks), and high-density sowing of chickpeas significantly reduced the FBNYV incidence [68] and may also be effective to control FBNYV virus infection in chickpeas in Egypt. In conclusion, integrated virus disease management measures are quite effective in the control of chickpea virus.

## 5. Summary and Prospect

Currently, there are 12 major viruses that infest chickpea and are transmitted by leafhoppers or aphids, causing symptoms such as yellowing and greening, stunting, and pod stunting, which cause severe losses. Proper detection and identification of chickpea viruses using various molecular diagnostic tools can accurately assess chickpea virus infection and provide a basis for chickpea virus disease control. To reduce the damage of chickpea virus disease, chickpea virus disease control measures should be developed according to local conditions, including chickpea disease resistance improvement, virus transmission pathways reduction, and integrated virus management, so as to reduce the economic losses caused by virus infection.

## 6. Discussion and Future Perspective

In recent years, the area planted with chickpea in China as well as the risk of occurrence and spread of chickpea virus disease has been increasing, and scientific prevention and management are urgently needed to avoid serious yield loss. Xinjiang is the largest area of chickpea cultivation in China, effectively promoting the realization of precise poverty alleviation in the region. Prevention and control of chickpea virus disease is an important measure to guarantee a good chickpea harvest; therefore, specific research on chickpea virus disease species identification, detection means, transmission routes, and virus transmission should be strengthened to develop specific and effective chickpea virus control measures in Xinjiang and safeguard chickpea cultivation.

## Figures and Tables

**Table 1:** Virus species of the family Luteoviridae and their geographical distribution.

Virus species	Geographical distribution	References
Chickpea chlorotic stunt virus, CpCSV	Algeria, Azerbaijan, Egypt, Eritrea, Ethiopia, Iran, Lebanon, Morocco, Syria, Tunisia, Yemen	[1, 11]
Bean leafroll virus, BLRV	Universal worldwide	[11]
Beet western yellows virus, BWYV	Universal worldwide	[11]
Soybean dwarf virus, SbDV	Australia, Ethiopia, Iran, Iraq, Japan, Syria, Tunisia, Uzbekistan	[11–13]
Cotton leafroll dwarf virus, CLRDV	Uzbekistan	[11]
Cucurbit aphid-borne yellows virus, CABYV	Sudan, Turkey	[11]
Phasey bean mild yellows virus, PhBMYV	Australia	[14]
Chickpea chlorotic dwarf virus, CpCDV	Middle East, Africa, South Africa, North Africa, South Asia, and Arabian Peninsula	Horn et al.; [12, 15–25]
Chickpea chlorosis virus, *CpCV*	New South Wales	[15, 26]
Chickpea redleaf virus, CpRLV	New South Wales	[16, 27]
Chickpea yellows virus, CpYV	Australia	[12, 16–25]
Mastrevirus	Australia	[12, 16–25]
Faba bean necrotic yellows virus, FBNYV	Syria	[28]
Cucumber mosaic virus, CMV	Temperate and tropical regions	[29, 30]
Alfalfa mosaic virus, AMV	Temperate and tropical regions	[29, 30]

**Table 2:** Comparison of the sensitivity of three plant virus detection methods.

Virus species	RT-PCR (m/fg)	IC-PCR (m/fg)	ELISA (m/pg)
ApMV	200	200	2000
PDV	2	20	2000
PNRSV	20	20	200
GFLV	20	200	200
CLRV	2	200	2000

## Data Availability

No data were used to support this study.
